# Age Cutoff and Yield of Prompt Esophagogastroduodenoscopy to Detect Malignancy in Vietnamese with Upper Gastrointestinal Symptoms: An Endoscopic Database Review of 472,744 Patients from 2014 to 2019

**DOI:** 10.1155/2021/1184848

**Published:** 2021-12-11

**Authors:** Duc Trong Quach, Lan Thi-Ngoc Tran, Truc Le-Thanh Tran, Vy Ly-Thao Tran, Nhan Quang Le, Toru Hiyama

**Affiliations:** ^1^Department of Internal Medicine, University of Medicine and Pharmacy at Hochiminh City, Ho Chi Minh City, Vietnam; ^2^Department of Endoscopy, University Medical Center at Hochiminh, Ho Chi Minh City, Vietnam; ^3^Department of Gastroenterology, An-Binh Hospital, Ho Chi Minh City, Vietnam; ^4^Health Service Center, Hiroshima University, Higashihiroshima, Japan

## Abstract

**Background and Aims:**

Age cutoff is an important factor in deciding whether esophagogastroduodenoscopy (EGD) is necessary for patients presenting with upper gastrointestinal symptoms. However, the cutoff value is significantly different across populations. We aimed to determine the age cutoff for EGD that assures a low rate of missing upper gastrointestinal malignancy (UGIM) and to assess the yield of prompt EGD in Vietnamese patients presenting with upper gastrointestinal symptoms.

**Methods:**

All EGDs performed in outpatients during a 6-year period (2014–2019) at a tertiary hospital that provided an open-access endoscopy service were retrospectively reviewed. Repeat or surveillance EGDs were excluded. Different age cutoffs were evaluated in terms of their prediction of the absence of UGIM. The yield of endoscopy to detect one malignancy (YoE) was also calculated.

**Results:**

Of 472,744 outpatients presenting with upper gastrointestinal symptoms, there were 2198 (0.4%) patients with UGIM. The median age and male-to-female ratio of patients with UGIMs were 57.9 ± 12.5 years and 2.5 : 1, respectively. The YoEs in patients <40, 40–60, and >60 years of age were <1, 1–10, and >10 per 1000 EGDs, respectively. The age cutoffs of 30 years in females and 35 years in males could detect 98.2% (95% CI: 97.7%–98.8%) of UGIM cases with a YoE of about 1 per 1000 EGDs.

**Conclusions:**

The age cutoff for EGD in Vietnamese should be lower than that recommended by current international guidelines. The strategy of prompt EGD showed a low YoE, and its cost-effectiveness requires further investigation.

## 1. Introduction

Upper gastrointestinal symptoms are prevalent worldwide. One of the main concerns in managing patients with these symptoms is the risk of missing upper gastrointestinal malignancy (UGIM). Esophagogastroduodenoscopy (EGD) is the most important investigation for detecting UGIM, but it is invasive and may be not cost-effective in some clinical settings. Although there are specific guidelines for dyspepsia and gastroesophageal reflux disease, the overlap of symptoms is very common in real-world practice [1]. All of these guidelines recommend using alarm features to identify patients who have priority for prompt performance of EGD [2–5]. However, studies from both Eastern and Western countries showed that the alarm features had very low sensitivity for UGIM [6, 7]. Age cutoff, therefore, is recommended as an adjunctive factor to avoid missing UGIM. The age cutoff for the selection of patients who should undergo EGD, however, varies significantly across the guidelines depending on the prevalence of UGIM in each population. Two meta-analysis studies have shown that the age at onset of UGIM in Asian populations is much lower than that in the West [8, 9]. However, the Asian data in these two meta-analyses were mainly based on studies conducted on Chinese populations [8, 9]. As the risk level of UGIM is significantly different across Asian populations [10, 11], further data from other Asian populations are necessary to clarify this issue.

Vietnam has the highest prevalence of gastric cancer among the Southeast Asian countries (ASEAN) with age-standardized incidence rates (ASR) per 100,000 of 23.7 in males and 10.8 in females [12, 13]. As in many other countries in the region, a strategy of prompt endoscopy (i.e., EGDs are not restricted to patients with alarm features or those above a specific age cutoff) has been applied popularly in daily practice in the management of upper gastrointestinal symptoms. However, the optimal cutoff age and the yield of prompt endoscopy for UGIM have not yet been determined.

This study was conducted to determine the cutoff age for EGD that would assure a low rate of missing UGIM and to assess the yield of prompt EGD to detect UGIM in Vietnamese patients with upper gastrointestinal symptoms.

## 2. Materials and Methods

### 2.1. Setting

This retrospective study was conducted at the University Medical Center at Hochiminh City, which is one of the two largest tertiary hospitals in the Southern region of Vietnam. The Department of Endoscopy of the hospital provides an open-access service so that general practitioners can directly refer their patients without prior specialist consultation. A prompt endoscopy approach has been applied extensively and is preferred to empiric treatment for the investigation of upper gastrointestinal symptoms due to patient preference and the low cost of service. The cost of an EGD procedure under local anesthesia was 800,000 Dong (34.64 USD), whereas that for a ^13^C breath test was 823,000 Dong (35.64 USD). There are no screening programs for UGIM at the hospital.

### 2.2. Data Retrieval Procedure

The computerized database of the hospital was used to retrospectively identify all EGDs performed between January 2014 and December 2019. All of these endoscopic procedures were performed by attending physicians using standard endoscopes (model GIF-H170 or GIF-H180, Olympus Corp., Tokyo, Japan). This database consists of inpatient and outpatient subcategories, but only the outpatient data were selected to calculate the total number of EGDs and to search for UGIMs with histopathologic confirmation. Patients who were not Vietnamese were excluded, and patients who underwent repeat endoscopy were counted only once each year during the study period. UGIM was defined as having any malignant lesions in the esophagus, stomach, or duodenum. In patients with confirmed UGIMs, all follow-up endoscopies were excluded to obtain the true number of new UGIM cases. The age and sex of all patients were recorded. The endoscopic and histopathologic characteristics of all UGIMs were extracted by trained physicians (Tran L., Tran T., Tran V., and Le N.) according to a predesigned questionnaire. The endoscopic types of esophageal and gastric cancers were reported according to the classifications of the Japanese Esophageal Society and the Japanese Gastric Cancer Association, respectively [14, 15], and the histopathologic findings were reported according to the World Health Organization classification of tumors of the digestive system [16]. Different age cutoffs were evaluated in terms of their prediction of the absence of UGIM. The yield of endoscopy to detect one malignancy per 1000 EGDs (YoE) was also calculated. The study protocol conforms to the ethical guidelines of the 1975 Declaration of Helsinki. This study was approved by the Board of Ethics in Biomedical Research of the University of Medicine and Pharmacy at Hochiminh City, Vietnam (number 342/HDDD-DHYD, signed on May 4, 2021).

### 2.3. Statistical Analysis

Categorical data are presented as numbers and percentages with 95% confidence interval (CI) and were analyzed using Pearson's chi-squared test. Quantitative data were tested for normality using the Kolmogorov–Smirnov test; and those with nonnormal distribution were presented as the median and interquartile range (IQR) and were compared using the Mann–Whitney *U* test. A *p* value <0.05 was considered statistically significant. All statistical analyses were carried out with SPSS 23 (SPSS Inc., Chicago, IL).

## 3. Results

**Figure 1:**
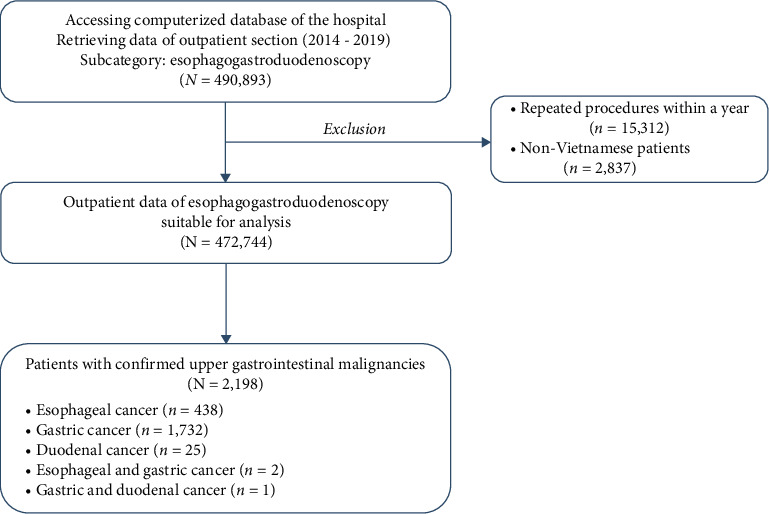
Data retrieval procedures of the study.

In total, 472,744 patients with EGDs performed during the period 2014–2019 fulfilled the criteria and were used for analysis (Figure 1). The male-to-female ratio was 1 : 1.2, and the median age was 42 (34–52) years. There were 2198 (0.4%) patients with UGIM, which included 438 (19.9%) esophageal cancers, 1732 (78.8%) gastric cancers, 25 (1.1%) duodenal cancers, and 3 (0.1%) concomitant UGIMs. The endoscopic and histopathologic characteristics of the UGIMs are presented in Table 1. The median age of the patients with UGIMs and esophageal, gastric, and duodenal malignancies was 57 (50–66), 57 (51–65), 57 (49–66), and 57.5 (49–63) years, respectively. The male-to-female ratio of the patients with UGIMs was 2.5 : 1. There were no significant differences in the age and sex distributions of the patients with UGIMs over the 6-year study period (Table 2).

One-hundred-forty-five patients with UGIMs were <40 years of age, and they accounted for 6.6% (CI 95%, 5.5%–7.6%) of all patients with UGIMs (Table 3). Of these patients, 138 (95.2%) were those with gastric cancer. The female proportion in this subgroup of patients was significantly higher compared to that in patients aged ≥40 years (53.8% (78/145) vs. 26.3% (540/2053), respectively, *p* < 0.01).

The proportion of UGIM at age cutoffs set at 30, 35, 40, 45, 50, and 55 years is presented in Table 3. The YoEs in the subgroups of patients <40, 40–60, and >60 years of age were <1, 1–10, and >10, respectively (Table 4).

## 4. Discussion

To our knowledge, this is the first large-scale report of age cutoff and yield of prompt endoscopy for Vietnamese patients with UGIMs, which includes all types of malignancies of the esophagus, stomach, and duodenum. The median age of the patients with UGIM was 57 (50–66) years, which did not significantly change during the 6-year study period. Most of the UGIM lesions showed endoscopically advanced features. Females were predominant among patients aged <40 years, in whom gastric cancer accounted for 95.2% of all UGIM cases. The cumulative frequencies of UGIM in patients aged <35, 40, and 45 years were 3.3, 6.6, and 13.7%, respectively. The YoE gradually increased with the advancement of age.

A recent systematic review showed that the YoE values for the detection of gastric and esophageal cancer were reported separately in several original studies [9]. However, as the overlap of upper gastrointestinal symptoms is very frequent in real-life practice [17], the YoE for all types of UGIM should be considered together. In the abovementioned systematic review, apart from studies in Africa which generally showed that esophageal cancer was the most predominant UGIM and gastric cancer accounted for less than half of all UGIMs, almost all large-scale studies in the West and in Asia consistently showed that gastric cancer accounted for more than two-thirds and up to 80% of all UGIM cases [9]. Duodenal cancer was very rare and only reported in a few large-scale studies. A previous study in China of 102,665 Chinese patients presenting with dyspepsia reported that the frequency of duodenal cancer was 0.7% of all UGIMs [7].

The age cutoffs for optimal detection of UGIM varied significantly across the published studies. Early-onset UGIM was very rare in the West [18, 19]. Consequently, prompt endoscopy conferred only a small benefit in terms of curing dyspepsia and was not a cost-effective strategy [20]. Current guidelines in Western countries recommend that the age cutoff for EGD in dyspepsia be 60 years [4, 5]. By contrast, patients with UGIM are much younger in Asia. A recent meta-analysis reported that the age cutoff should be 50 years of age in South America and Asia and 55 years of age in North America and Europe to detect at least 80% of UGIM in patients with upper gastrointestinal symptoms [9]. In another meta-analysis, the frequencies of gastric cancer under the ages of 45 years and 35 years in Asia were 17.8% and 3.0%, respectively [8]. Therefore, the authors suggested that the age cutoff of 35 years be applied to avoid missing UGIM in patients with uninvestigated dyspepsia in Asia [8]. However, the prevalence of gastric cancer, the most common type of UGIM in Asia, varies significantly across Asian populations [12, 13]. The 2012 Asian consensus on the management of functional dyspepsia recommended the cutoff ages of 40, 45, and 50 years for investigating dyspepsia in populations with high, intermediate, and low prevalence of gastric cancer, respectively [2]. There have been no updated versions of this consensus to date. Vietnam is a country with a high prevalence of gastric cancer, for which the ASR is 23.7 per 100,000 [12]. In a previous study, we reported a high percentage of early-onset gastric cancer in Vietnamese whose alarm features had a sensitivity of only 60.8% [21]. Therefore, an aggressive approach must be applied to not miss UGIM, especially gastric cancer, in young Vietnamese patients with upper gastrointestinal symptoms. The present study showed that applying an age cutoff of 40 years according to the Asian consensus would lead to a UGIM miss rate of 6.6% in Vietnamese. When stratified by sex, the frequencies of UGIM in the subgroup of patients aged <40 years were 12.6% in females and 4.2% in males. The age cutoff, therefore, must be lower in females than males in the Vietnamese. In a similar study in Taiwan, Liou et al. [22] did not find a difference in age cutoff between the two sexes in patients <60 years of age with gastric cancer. However, a recent large-scale study of 70,084 patients with gastric cancer showed that females were predominant in the subgroup of patients aged <40 years [23]. Hormonal factors were suggested to be associated with this finding, but a clear explanation remains unresolved [24].

The YoE for UGIM is an essential index when considering whether prompt endoscopy is an affordable approach in patients with upper gastrointestinal symptoms as it directly relates to cost-effectiveness and endoscopy workload. The YoE for UGIM varies significantly across previous studies, ranging from 3 to 137 per 1000 EGDs [9]. This could be explained by the differences in patient selection across the studies and the local prevalence of UGIM in the target populations. Some studies assessed the YoE in patients with alarm features or with advanced age, whereas others assessed the YoE based on a prompt endoscopy strategy [7, 9, 22]. A large-scale study in Taiwan, a region with an intermediate risk of gastric cancer (ASR: 18.6 and 10.5 per 100,000 in males and females, respectively), reported a YoE for gastric cancer that was slightly higher than that in our study (7.0 vs. 4.6 per 1000 EGDs) [22]. The proportion of patients aged <40 years in the Taiwan study was much lower than that in our study (5.8% vs. 41.7%, respectively), which would explain the lower YoE in our study as the prevalence of UGIM is generally higher in patients with advanced age.

Which age cutoff is optimal for prompt endoscopy is a clinically important question. The management of patients with upper gastrointestinal symptoms should aim to minimize the risk of missing UGIM. However, several factors should be considered when deciding on the most appropriate strategy in real-life practice: a cost-effectiveness analysis, patient preference, and local medical resources. Although the Asian consensus recommends that the age cutoff for EGD should be tailored according to the prevalence of UGIM in each population, recent studies showed that the frequency of early-onset UGIM does not always parallel with the ASR of UGIM in each population. For example, the ASR of gastric cancer in China is higher than that in Vietnam, but a large-scale study in China showed that the frequency of gastric cancer in patients aged <35 years was only 0.6% (95% CI, 0.5%–0.7%), which was significantly lower than that in our study [7, 12]. The frequency of early-onset UGIM, therefore, should also be considered when determining the appropriate age cutoff in each population.

Our study, in line with the meta-analysis by Chen et al. [8] in Asia, found a frequency of 3.3% (95% CI, 2.6%–4.1%) of UGIM at the age cutoff of 35 years. The optimal age cutoff in Vietnamese would thus be 30 years in females and 35 years in males for several reasons. First, there was only a 0.9% rate of UGIM in the subgroup of patients aged <30 years, which accounted for 14.9% of the study sample. A prompt endoscopy strategy had a YoE of only 0.3 per 1000 EGDs while significantly increasing the endoscopy workload in this age range. However, the cumulative frequency of UGIM increased abruptly from 0.9% to 3.3% at the age cutoffs of 30 and 35 years, respectively. Second, our study showed that applying this age cutoff might reduce the rate of missing UGIM to 1.8% in both sexes with a YoE of ≥1 per 1000 EGDs. Other Asian studies suggested age cutoffs for EGD with almost the same YoE. In Taiwan, Liou et al. [22] reported that an age cutoff of 40 years in patients with simple dyspepsia might lead to a rate of missing UGIM of 6.1% with a YoE of 1.02 per 1000 EGDs. In Singapore, Wai et al. [25] reported that an age cutoff of 45 years led to a rate of missing UGIM of 12.0% with a YoE of 1.15 per 1000 EGDs. Third, Vietnamese patients with upper gastrointestinal symptoms tend to prefer endoscopy to a noninvasive approach due to their concern about having gastric cancer. Although further cost-effectiveness analysis is required, the low cost and wide availability of the EGD procedure in Vietnam are great advantages that make a prompt EGD strategy easy to extend to such a low age cutoff. In Western countries, by contrast, the cost for EGD is much higher and prompt endoscopy strategy might be cost-effective only in patients over 50 years of age [20, 26]. Fourth, the majority of UGIM lesions in our study were detected in an endoscopically advanced stage and were not curable by endoscopic resection techniques. As the median progression time of gastric cancer from early stage to advanced stage was reported to be about 34 to 44 months [27, 28], choosing this age cutoff would lead both to the increased detection of UGIMs in their earlier stages and to a better survival prognosis. Finally, precancerous gastric lesions were prevalent in Vietnamese patients with upper gastrointestinal symptoms [29]. Even in the absence of UGIM, performing EGD will provide an opportunistic screening and help to identify patients with high-risk precancerous lesions for gastric cancer surveillance [30].

Currently, there have been no national screening programs for gastric cancer in Vietnam, and most patients with gastric cancer were diagnosed in the advanced stage with a very low 5-year survival rate [13]. Given the high prevalence of gastric cancer and the low sensitivity of alarm features in Vietnamese, there is an urgent need for a population-based screening program. Besides some clinical characteristics which have been documented as high-risk factors of gastric cancer such as male gender, smoking status, family history of gastric cancer, and *H. pylori* infection [30], this study provides a reference on the age cutoff to develop such a program.

This study has some limitations. First, this is a retrospective single-center study, and details regarding patients' symptoms could not be reliably retrieved. However, as upper gastrointestinal symptoms often overlap, the results of this study indicated real-life practice. Second, alarm features could not be reliably recorded due to the retrospective design. Therefore, the cutoff age for EDG in the subgroup of patients with or without alarm features could not be evaluated separately. However, as the sensitivity of these features was reported to be unacceptably low in Vietnamese patients with upper gastrointestinal symptoms, we think that this would not have a great impact on the present results.

## 5. Conclusions

In conclusion, the age cutoff that assures a low rate of missing UGIM in Vietnamese was lower than that recommended by international guidelines, and it would be lower in females compared to males. Given the low cost of EGD, its widespread availability, and patient preference in Vietnam, prompt endoscopy would be a suitable approach for patients with upper gastrointestinal symptoms in the country. The optimal age cutoff would be 30 years in females and 35 years in males, which could help to detect 98.2% of UGIMs in both sexes with a YoE of about 1 per 1000 EGDs. This study also showed that the optimal age cutoff for EGD should be considered based not only on the prevalence of UGIM but also on the frequency of early-onset UGIM in each population.

## Figures and Tables

**Table 1:** Endoscopic and histopathologic characteristics of the upper gastrointestinal malignancies.

Endoscopic and histopathologic characteristics	*n* (%)
Esophagus (*n* = 440)	
Location, *n* (%)	
Cervical	14 (3.2)
Upper thoracic	60 (13.6)
Middle thoracic	136 (30.9)
Lower thoracic	188 (42.7)
Abdominal	42 (9.5)

Endoscopic type^1^, *n* (%)	
Type 0	1 (0.2)
Type 1	247 (56.1)
Type 2	153 (34.8)
Type 3	17 (3.9)
Type 4	18 (4.1)
Type 5	4 (0.9)

Pathologic type, *n* (%)	
Squamous cell carcinoma	375 (85.2)
Adenocarcinoma	49 (11.1)
Others	16 (3.7)

Stomach (*n* = 1735)	
Location, *n* (%)	
Upper part	109 (6.3)
Middle part	365 (21.0)
Lower part	975 (56.2)
≥ 2 parts involved	286 (16.5)

Endoscopic type^2^, *n* (%)	
Type 0	86 (5.0)
Type 1	186 (10.7)
Type 2	948 (54.6)
Type 3	301 (17.3)
Type 4	212 (12.2)
Type 5	2 (0.1)

Pathologic type, *n* (%)	
Adenocarcinoma	
Intestinal type	1250 (72.0)
Diffuse type	418 (24.1)
Lymphoma	59 (3.4)
Others	8 (0.5)

Duodenum (*n* = 26)	
Location, *n* (%)	
Superior duodenal flexure	3 (11.5)
Descending part	23 (88.5)

Endoscopic type, *n* (%)	
Mass	15 (57.7)
Ulcerative	9 (34.6)
Infiltrative ulcerative	2 (7.7)

Pathologic type, *n* (%)	
Adenocarcinoma	18 (69.2)
Secondary carcinoma (invasion or metastasis)	4 (15.4)
Lymphoma	2 (7.7)
Gastrointestinal stromal tumor	1 (3.8)
Neuroendocrine tumor	1 (3.8)

^1, 2^The endoscopic types of esophageal and gastric cancers were reported according to the classifications of the Japanese Esophageal Society and the Japanese Gastric Cancer Association, respectively.

**Table 2:** Age and sex distribution of the patients with upper gastrointestinal malignancy during the 2014–2019 period.

		Year of endoscopy and number of patients with upper gastrointestinal malignancy						Total *N* = 2198	*p* Value
		2014 *n* = 346	2015 *n* = 381	2016 *n* = 396	2017 *n* = 346	2018 *n* = 383	2019 *n* = 346		
Age	Median (IQR)	58 (50–67)	58 (50–67)	56.5 (48–66)	58 (51–67)	58 (50–65)	56 (51–63)	57 (50–66)	0.399
	Minimum	23	14	26	20	28	20	14	—
	Maximum	90	90	92	94	89	86	94	—

Age range	<40 (*n*, %)	22 (6.4)	24 (6.3)	24 (6.1)	24 (6.9)	28 (7.3)	23 (6.6)	145 (6.6)	0.985
	≥40 (*n*, %)	324 (93.6)	357 (93.7)	372 (93.9)	322 (93.1)	355 (92.7)	323 (93.4)	2053 (39.4)	

Sex	Female (*n*, %)	97 (28.0)	107 (28.1)	127 (32.1)	82 (23.7)	111 (29.0)	94 (27.2)	618 (28.1)	0.244
	Male (*n*, %)	249 (72.0)	274 (71.9)	269 (67.9)	264 (76.3)	272 (71.0)	252 (72.8)	1580 (71.9)	

IQR: interquartile range.

**Table 3:** Age cutoff of esophagogastroduodenoscopy according to the cumulative percentage of missed upper gastrointestinal malignancy.

Age cutoff (years)	Female *N* = 618		Male *N* = 1580		Total *N* = 2198	
	*n*	Cumulative % (95% CI)	*n*	Cumulative % (95% CI)	*n*	Cumulative % 95% CI)
≤30	11	1.8 (0.9–3.1)	9	0.6 (0.3–1.1)	20	0.9 (0.6–1.4)
≤35	44	7.1 (5.2–9.4)	28	1.8 (1.2–2.5)	72	3.3 (2.6–4.1)
≤40	78	12.6 (10.1–15.5)	67	4.2 (3.3–5.4)	145	6.6 (5.6–7.7)
≤45	132	21.4 (18.0–24.6)	169	10.7 (9.2–12.3)	301	13.7(12.3–15.2)
≤50	191	30.9 (27.3–34.7)	334	21.1 (19.2–23.2)	525	23.9 (22.1–25.7)
≤55	268	43.4 (39.4–47.4)	620	39.2 (36.8–41.7)	888	40.4 (38.3–42.5)

CI: confidence interval.

**Table 4:** Yield of esophagogastroduodenoscopy for upper gastrointestinal malignancy with prompt endoscopy strategy.

Age range (years)	Number of endoscopic procedures		Upper gastrointestinal malignancies	
	*N*	Proportions of procedures % (95% CI)	*N*	Yield per 1000 procedures % (95% CI)
<30	70 600	14.9 (14.8–15.0)	20	0.3 (0.2–0.4)
30– < 35	57 697	12.2 (12.1–12.3)	52	0.9 (0.7–1.2)
35– < 40	69 131	14.6 (14.5–14.7)	73	1.1 (0.8–>1.3)
40– < 45	67 318	14.2 (14.1–14.3)	156	2.3 (2.0–2.7)
45– < 50	61 660	13.0 (12.9–13.1)	224	3.6 (3.2–4.1)
50– < 55	52 808	11.2 (11.1–11.3)	363	6.9 (6.2–7.6)
55– < 60	40 036	8.5 (8.4–8.6)	359	9.0 (8.1–9.9)
60– < 65	26 807	5.6 (5.6–5.7)	324	12.1 (10.8–13.5)
65– < 70	14 079	3.0 (2.9–3.0)	258	18.3 (16.2–20.7)
≥70	12 608	2.6 (2.6–2.7)	369	29.3 (26.4–32.4)

CI: confidence interval.

## Data Availability

The data that support the findings of this study are available from the corresponding author upon reasonable request.
